# Evolution of the Exon-Intron Structure in Ciliate Genomes

**DOI:** 10.1371/journal.pone.0161476

**Published:** 2016-09-07

**Authors:** Vladyslav S. Bondarenko, Mikhail S. Gelfand

**Affiliations:** 1 Institute of Molecular Biology and Genetics, NASU, Zabolotnogo Str. 150, Kyiv, 03680, Ukraine; 2 A.A. Kharkevich Institute for Information Transmission Problems, RAS, Bolshoy Karetny per. 19, Moscow, 127994, Russia; 3 Skolkovo Institute of Science and Technology, Moscow, 143026, Russia; 4 Department of Bioengineering and Bioinformatics, M.V. Lomonosov Moscow State University, Vorobievy Gory 1–73, Moscow GSP-1, 119234, Russia; National Center for Biotechnology Information, UNITED STATES

## Abstract

A typical eukaryotic gene is comprised of alternating stretches of regions, exons and introns, retained in and spliced out a mature mRNA, respectively. Although the length of introns may vary substantially among organisms, a large fraction of genes contains short introns in many species. Notably, some Ciliates (*Paramecium* and *Nyctotherus*) possess only ultra-short introns, around 25 bp long. In *Paramecium*, ultra-short introns with length divisible by three (3n) are under strong evolutionary pressure and have a high frequency of in-frame stop codons, which, in the case of intron retention, cause premature termination of mRNA translation and consequent degradation of the mis-spliced mRNA by the nonsense-mediated decay mechanism. Here, we analyzed introns in five genera of Ciliates, *Paramecium*, *Tetrahymena*, *Ichthyophthirius*, *Oxytricha*, and *Stylonychia*. Introns can be classified into two length classes in *Tetrahymena* and *Ichthyophthirius* (with means 48 bp, 69 bp, and 55 bp, 64 bp, respectively), but, surprisingly, comprise three distinct length classes in *Oxytricha* and *Stylonychia* (with means 33–35 bp, 47–51 bp, and 78–80 bp). In most ranges of the intron lengths, 3n introns are underrepresented and have a high frequency of in-frame stop codons in all studied species. Introns of *Paramecium*, *Tetrahymena*, and *Ichthyophthirius* are preferentially located at the 5' and 3' ends of genes, whereas introns of *Oxytricha* and *Stylonychia* are strongly skewed towards the 5' end. Analysis of evolutionary conservation shows that, in each studied genome, a significant fraction of intron positions is conserved between the orthologs, but intron lengths are not correlated between the species. In summary, our study provides a detailed characterization of introns in several genera of Ciliates and highlights some of their distinctive properties, which, together, indicate that splicing spellchecking is a universal and evolutionarily conserved process in the biogenesis of short introns in various representatives of Ciliates.

## Introduction

Ciliates represent a unique and extremely heterogeneous group of protozoans containing many thousands of genetically diverse species [1]. Named after a hair-like organelle called cilia, they have a number of distinguishable features which make them important model organisms to study various biological phenomena such as RNA splicing, DNA rearrangements, and telomeres [2]. Unlike other eukaryotes, each ciliate possesses two types of nuclei, germline micronucleus (MIC) and somatic macronucleus (MAC) which derives from MIC during extensive developmental rearrangements of the genomic DNA. The diploid micronucleus serves as the germline source of DNA during reproduction and is transcriptionally silent, whereas the macronucleus expresses genes to support vegetative cell growth and cell proliferation [1]. Each nucleus contains distinct genomes, which are replicated, amplified, and eliminated at different stages of the ciliate life cycle [1–4].

The micronuclear DNA consists of extremely long molecules typical of eukaryotic chromosomes, containing genes interrupted by short non-coding elements, called internal eliminated sequences (IESs), and large stretches of the spacer DNA [2]. In contrast, the macronuclear DNA exists as short molecules ranging in size from a few hundred to several thousand base pairs (bp) with high gene density and without IESs and spacer DNA [2–4].

To date, macronuclear genomes of several ciliates have been completely sequenced and annotated (Class Oligohymenophorea, genera *Tetrahymena*, *Ichthyophthirius*, *Paramecium*; class Spirotrichea, genera *Oxytricha*, *Stylonychia*, *Urostyla*, *Paraurostyla*, *Laurentiella*) [5–10]. The MAC genome of *Euplotes crassus* (class Armophorea, genus *Nyctotherus*) had also been sequenced, but its annotation is not complete [11]. The numbers of genes as well as the general principles of the genome organization have been shown to be dramatically different between the organisms. The MAC genome of *Tetrahymena thermophila*, 104 MB in length, is comprised of 225 chromosomes and encodes almost 27000 genes [6]. During its differentiation it becomes 10–20% smaller than that of MIC, due to removal of the repetitive DNA and about 6000 IESs. The MAC genome of a closely related species, *Ichthyophthirius multifiliis*, is approximately three-fold smaller and has three-fold fewer genes (around 8000), which are significantly shorter than those of *Tetrahymena* [7]. These and other reductions have been suggested to occur due to adaptation of *Ichthyophthirius* to the parasitic lifestyle. On the contrary, the MAC genome of *Paramecium tetraurelia* has undergone numerous whole-genome duplications resulting in about 40000 genes with dozens of copies [5]. During MAC formation, around 45000 of IESs and other repetitive DNA are removed from MIC, which corresponds to ~25% of the MAC genome [5, 12]. Representatives of Spirotrichea, *Oxytricha trifallax* and *Stylonychia lemnae*, have a unique MAC genome organization comprised of thousands of so-called nanochromosomes, each typically encoding a single gene, and differently amplified up to ~2000 copies on average after extensive removal of ~96% of the MIC genome content [8]. Another distinctive feature of spirotricheans is a non-sequential or even reverse order of gene segments in the macronuclear and micronuclear DNA [2, 8]. The correct order and orientation of gene segments in the macronucleus are established during the process called genome unscrambling, which is guided by RNA [13].

The MIC-to-MAC genome reduction in Ciliates is also associated with fewer and smaller introns. In general, genomes with low intron densities also tend to have smaller introns and tend to be reduced in other ways as well, as seen for the yeast genome and more extremely compacted genomes such as those of microsporidia [14, 15]. The average number of introns per gene is low in Ciliates and varies from 1.6–2.3 in *Paramecium* and *Oxytricha* [5, 8] to 4.8 in *Tetrahymena* [7]. The alveolate ancestor was predicted to be relatively intron-rich with intron density comparable to that of human genes, indicating predominant introns loss during the evolution of alveolates [16]. For spirotricheans, one explanation of the observed introns scarcity could be transformation of introns into IESs leading to their permanent excision from the micronuclear genome during the MIC-to-MAC transition [17, 18]. However, the details of this mechanism in spirotricheans as well as its presence in other lineages remain unknown.

In *Paramecium*, the length of all introns lies in an extremely narrow range, 25–45 bp [5]. These are among the smallest introns known, likely reflecting a functional limit for spliceosomal recognition [19]. The intron size limitations for short introns recognition and exclusion from pre-mRNA are likely to be compensated by splicing spellchecking mechanisms such as nonsense-mediated decay (NMD). Indeed, in *Paramecium*, 3n introns (with length divisible by three) are under strong evolutionary pressure and have a high frequency of in-frame stop codons [20]. In the case of introns retention, the latter would guarantee termination of the translation of mis-spliced mRNAs and, hence eliminate production of potentially misfolded proteins.

**Fig 1:**
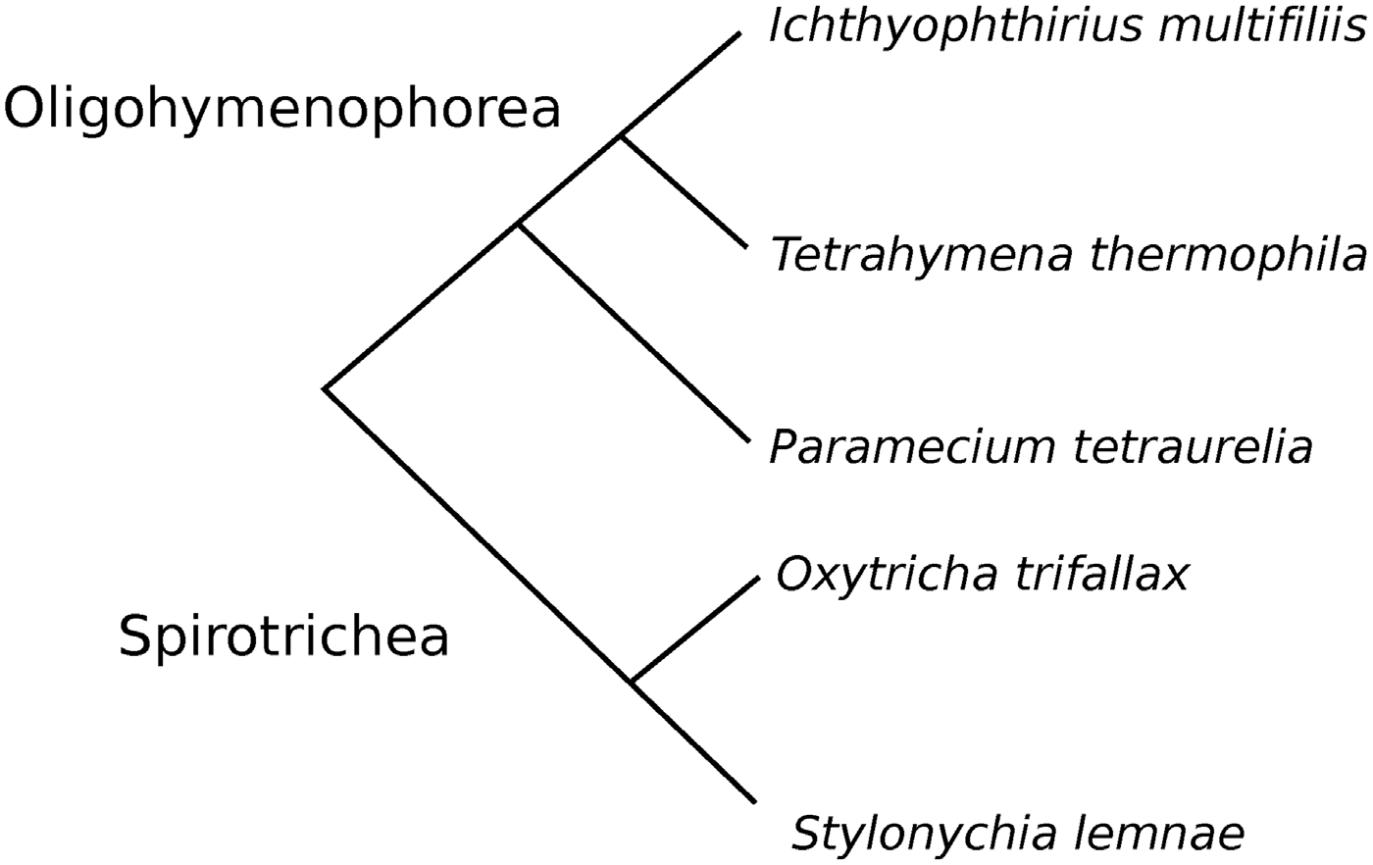
Phylogenetic relationships between the studied species.

In many species, a substantial fraction of genes contains short introns [21]. From this perspective, Ciliates provide a useful model to explore the mechanisms of splicing and the evolution of short introns, as either all or most of their introns are very short [5–10]. Here, we considered introns in the genomes of several representatives of Ciliates, *Paramecium*, *Tetrahymena*, *Ichthyophthirius*, *Oxytricha*, and *Stylonychia* (Fig 1). In each organism, we studied intron lengths, abundance, and distributions of 3n and 3n±1 introns, frequencies of in-frame stop-codons in introns, their splice sites, and evolutionary conservation of the introns positions and lengths.

## Results

### Characterization of short introns in *Paramecium*, *Tetrahymena*, *Ichthyophthirius*, *Oxytricha*, and *Stylonychia*

**Fig 2:**
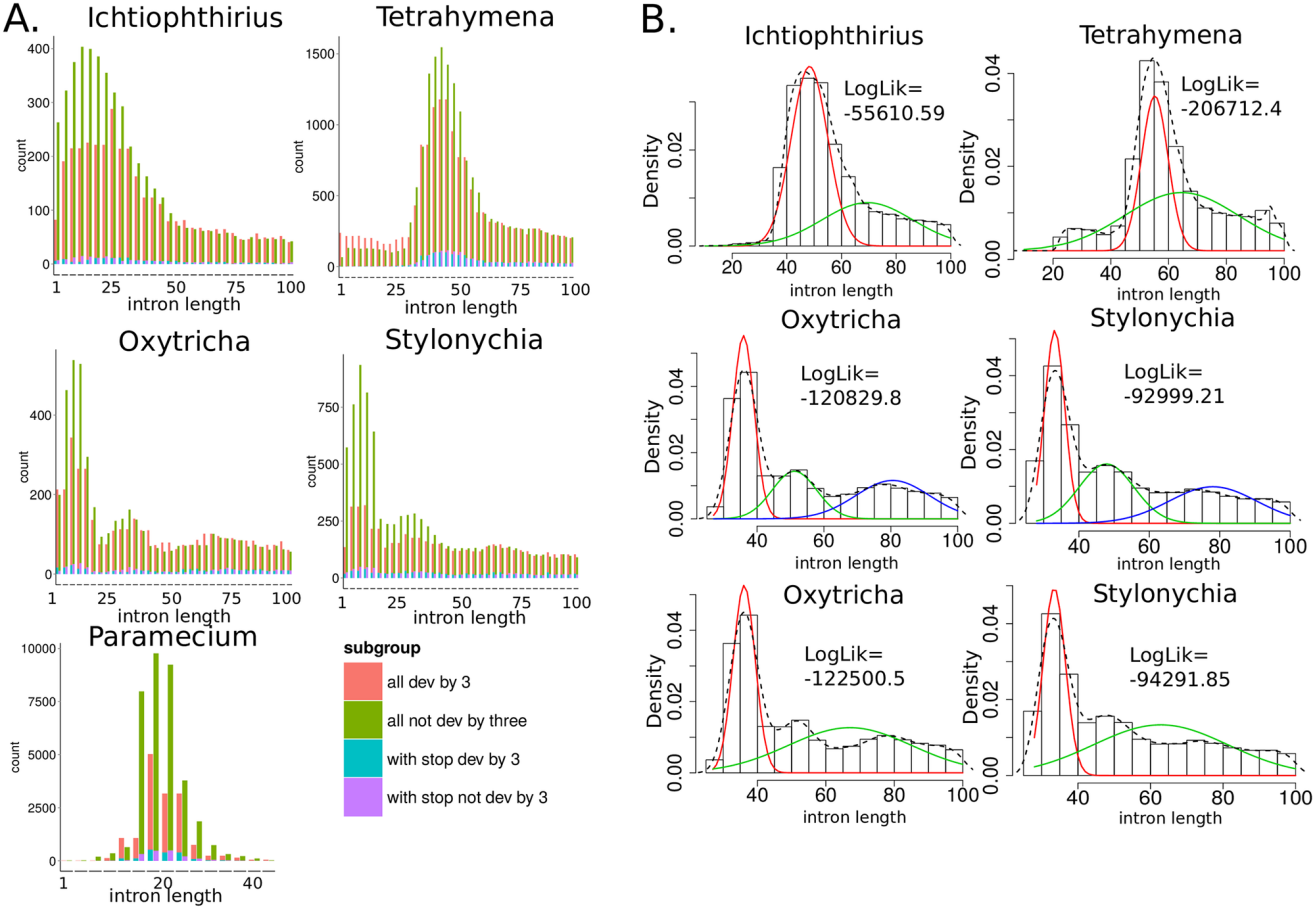
**A. Intron length distributions of *Paramecium*** [5], ***Tetrahymena*** [6], ***Ichthyophthirius*, *Oxytricha*** [8], **and *Stylonychia***. Introns containing “n” or “N” nucleotides were excluded. Pink and green colors show the numbers of 3n±1 and 3n introns, respectively. Blue and purple colors show the numbers of 3n±1 and 3n introns with in-frame stop codons, respectively. **B. Decomposition of the intron length distributions of *Tetrahymena*, *Ichthyophthirius*, *Oxytricha*, and *Stylonychia* in the length interval 1–100 bp into a sum of 2 or 3 weighted normal distributions**. Histograms show the observed distributions without decomposition, whereas red, blue, and green curves denote the decomposed weighted normal distributions.

**Fig 3:**
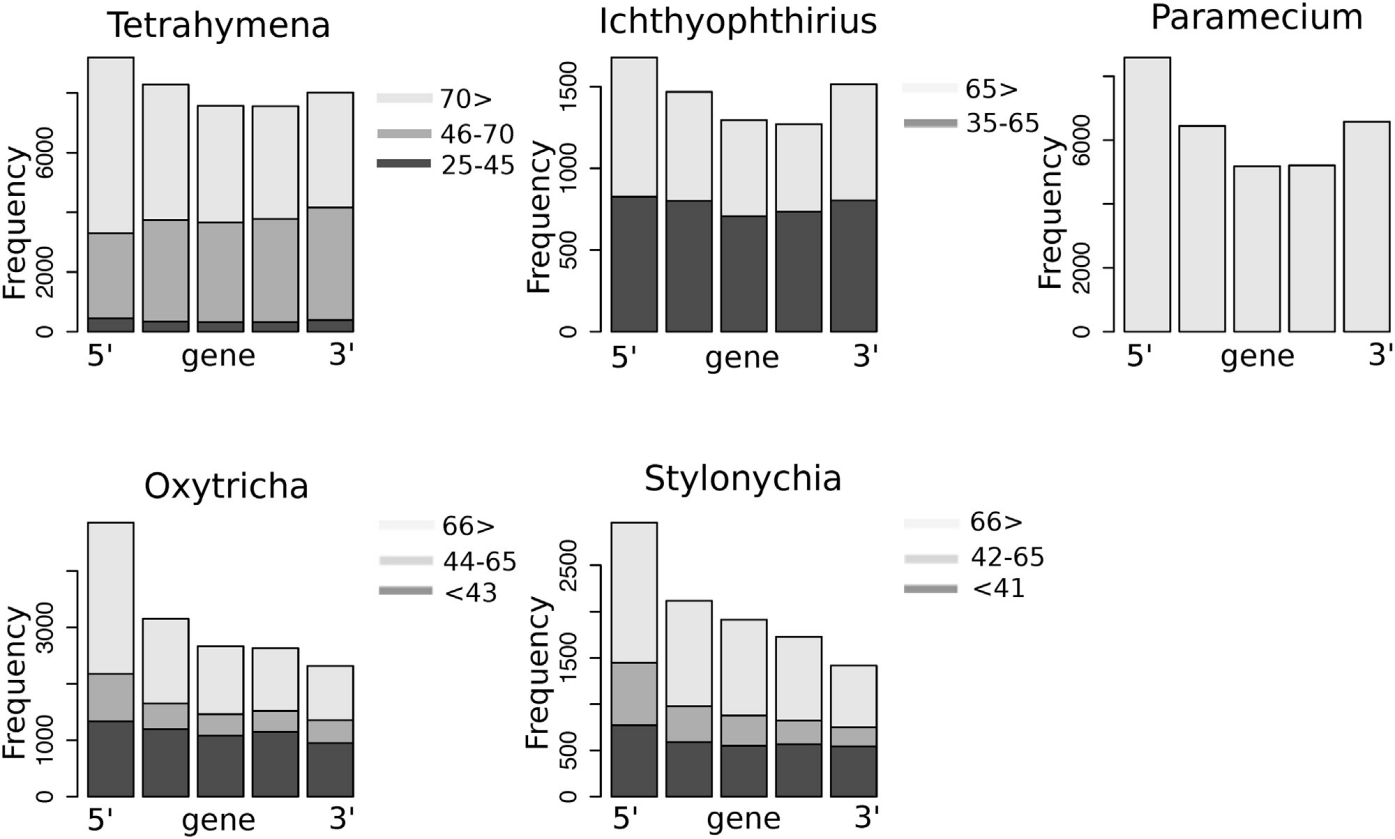
Densities of introns along the genes. Genes containing at least five introns were selected and divided into five equal intervals; then numbers of introns in each interval were calculated. Different bar colors designate groups of introns (see the inserted legend). The same trend was observed for genes with ≥ 3 introns (S4 Fig).

Fig 2 features the distributions of intron lengths in genes of *Paramecium*, *Tetrahymena*, *Ichthyophthirius*, *Oxytrich*a, and *Stylonychia*. Intron lengths of three closest species, *Paramecium*, *Tetrahymena*, and *Ichthyophthirius*, have unimodal distributions with modes 25 bp, 56 bp, and 48 bp, respectively. In the interval of 1–100 bp, the intron length distributions of *Ichthyophthirius* and *Tetrahymena* can be decomposed into two weighted normal distributions (Fig 3), which likely correspond to two different intron length classes with means 48 bp, 69 bp, and 55 bp, 64 bp, respectively. Interestingly, in the same length interval, the distributions of *Oxytricha* and *Stylonychia* intron lengths have three modes [8] and can be represented as a mixture of three normal distributions with means 33–35 bp, 47–51 bp, and 78–80 bp (Table 1). Decomposition of these distributions into a sum of two weighed normal distributions results in a worse approximation with a significantly lower log-likelihood (differences in the Akaike information criteria values between the 3-component and the 2-component mixture models equal −3190 and −2580 for *Oxytricha* and *Stylonychia*, respectively), indicating that these organisms possess three distinct classes of introns.

**Table 1:** Parameters of the weighted normal decomposition components of the introns length distributions of *Ichthyophthirius*, *Tetrahymena*, *Oxytrich*a, and *Stylonychia* in the introns length interval 1–100 bp.

Parameter	First component	Second component	Third component	Organism
Mean	48.3	69.5	–	*Ichthyophthirius*
SD	6.7	16.5	–	
Weight	0.6	0.4	–	
Log-likelihood	−55611			
Mean	55.2	64.0	–	*Tetrahymena*
SD	4.6	19.5	–	
Weight	0.4	0.6	–	
Log-likelihood	−206712			
Mean	36.0	51.2	80.5	*Oxytricha*
SD	3.2	6.6	11.0	
Weight	0.4	0.2	0.3	
Log-likelihood	−120830			
Mean	33.1	47.8	78.1	*Stylonychia*
SD	2.9	7.8	12.5	
Weight	0.4	0.3	0.3	
Log-likelihood	−92999			

From the intron length distributions in Fig 2 it is clear that 3n introns are distributed differently in all studied species. We observed that 3n introns are significantly underrepresented in 33–63 bp introns of *Ichthyophthirius*, 45–62 bp introns of *Tetrahymena*, 27–69 bp introns of *Stylonychia*, and 27–38 bp introns of *Oxytricha* (as described in Methods, “Calculation of 3n introns underrepresentation”). Therefore, 3n introns depletion was intrinsic for short introns of all studied species, whereas no significant under-representation of 3n introns was identified for longer introns. Interestingly, in *Tetrahymena*, 3n introns in the range 22–44 bp were significantly more frequent, and, relative to 3n±1 introns, depleted in in-frame stop codons (Table 2).

**Table 2:** Depletion of 3n introns in *Ichthyophthirius*, *Tetrahymena*, *Oxytricha*, and *Stylonychia*.

Introns length interval, bp	Organism	Over- or underrepresentation of 3n introns in the length interval	P-value
33–63	*Ichthyophthirius*	1.4-fold underrepresented	< 2.2x10^-16^
45–62	*Tetrahymena*	1.3-fold underrepresented	< 2.2x10^-16^
22–44	*Tetrahymena*	1.5-fold overrepresented	< 2.2x10^-16^
27–38	*Oxytricha*	1.3-fold underrepresented	< 2.2x10^-16^
27–69	*Stylonychia*	1.7-fold underrepresented	< 2.2x10^-16^

In order to analyze densities of introns along the genes with respect to the introns length, we divided introns into several discrete groups by their length. For *Tetrahymena* and *Ichthyophthirius*, the groups were defined based on the inflection points of the intron length densities and comprised of 25–45 bp (ultra-short), 46–70 bp (short), and >70 bp (long) groups for *Tetrahymena*, and 35–65 bp (short) and >65 bp (long) groups for *Ichthyophthirius*. For *Oxytricha* and *Stylonychia*, the groups were <43 bp (ultra-short), 44–65 bp (short), and >66 bp (long), as defined based on the local minima of the fitted introns length distributions.

Intron positions in *Oxytricha* and *Stylonychia* were strongly skewed towards the 5' end of a gene, especially in the groups of short and long introns (Fig 3). Interestingly, none of the intron groups of *Tetrahymena* and *Ichthyophthirius* are uniformly distributed along the genes, but peaked at their 5' and 3' ends to a different extent. The pattern is most distinct in *Paramecium* and less so in *Tetrahymena*, although long introns of *Tetrahymena* tend to be more non-evenly distributed than short introns (Fig 3). *Tetrahymena* is most intron-rich among the studied organisms, and its introns density across the genes most closely resembles the uniform distribution, whereas *Paramecium* has almost twofold fewer introns per gene.

### Splice sites in short introns

The initial stage of splicing, precise introns recognition, depends on a complex organization of regulatory motifs, which interact with different parts of the spliceosomal complex and are located both in introns and exons [22]. However, although short introns prevail in the studied genomes, it is not clear how these extremely small introns are recognized and processed by the spliceosomal machinery [15]. Therefore, we first explored the nucleotide composition at the 5' and 3' splice sites.

**Fig 4:**
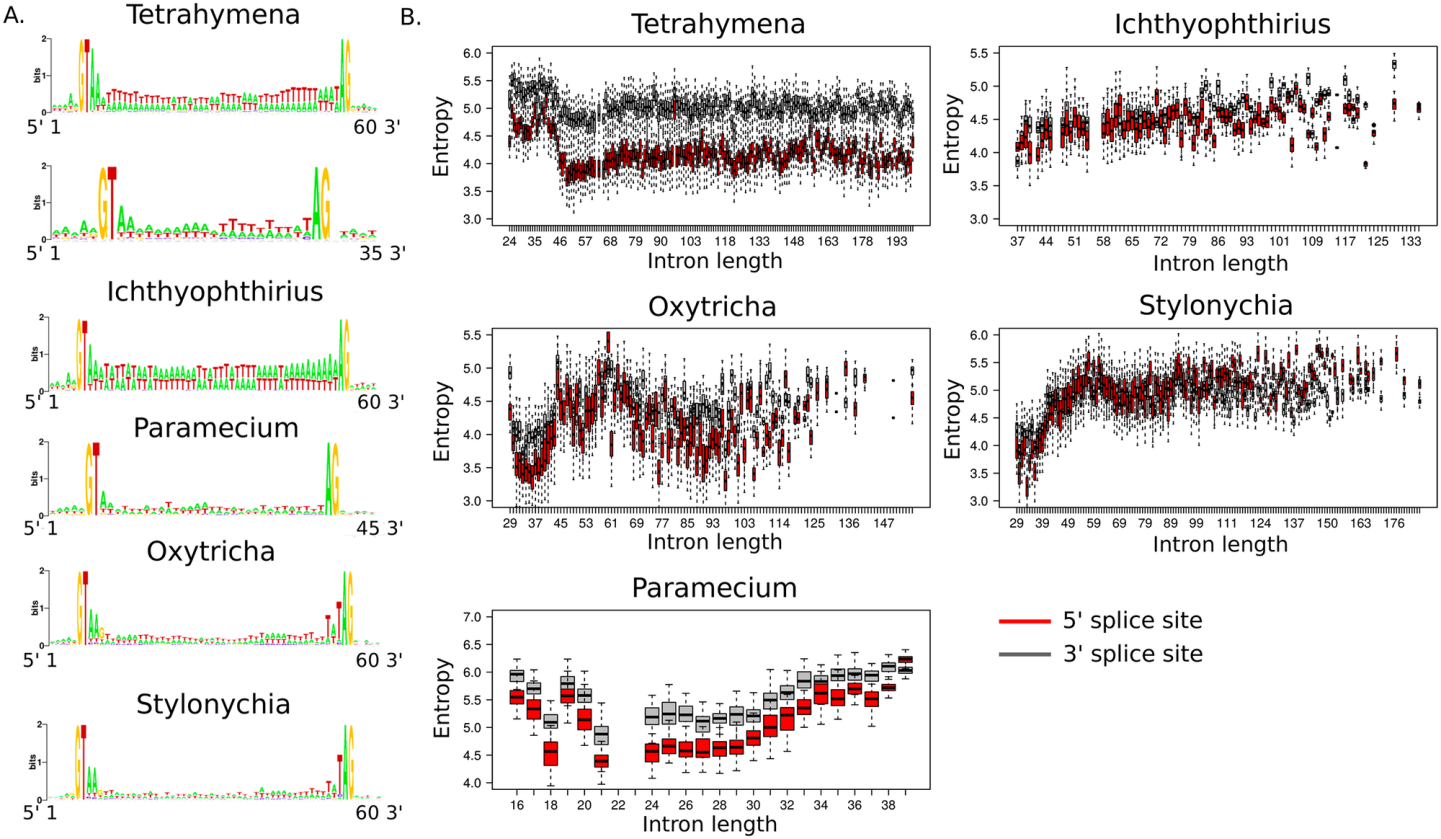
**A. Conserved nucleotides at 5' and 3' ends of introns of *Tetrahymena*, *Ichthyophthirius*, *Paramecium*, *Oxytricha*, and *Stylonychia*** [5–10]. For each intron, a 5 bp long exon part was also considered. **B. Strength of the 5' and 3' splice site motif as a function of the intron length**. The strength of the splice site motif was calculated as the entropy of its frequency matrix, see the main text. Red and black boxplots designate the entropy measure of 5' and 3' splice sites, respectively. In order to account for uneven sample size, for each intron length up to 200 bp, we randomly sampled 50 introns 50 times and estimated their average splice sites entropy. Intron length intervals with less than 50 introns were excluded.

Like all eukaryotes, Ciliates have conserved dinucleotides at the intron termini [5–10], hence satisfying the GT—AG, or Breathnach-Chambon, rule [23]. However, introns of *Oxytricha* and *Stylonychia* have longer splice motifs with five conserved nucleotides at both ends (5'–GTAAG…TATAG–3') [8, 9, 24] (Fig 4A). Exon regions adjacent to the exon-intron boundaries are not conserved in any of the studied species.

We analyzed the relation between the strength of the 3' and 5' splice site motifs and the intron length. For this purpose, we considered 5 bp-long 5' and 3' boundaries of each intron, constructed frequency matrices, and calculated the entropy of the obtained matrices by the formula:

$$Entropy=-\underset{i,j}{\sum }p_{i,j}\cdot log\left(p_{i,j}\right)$$

where *p*_*i*,*j*_ is the frequency of a nucleotide *i* at a motif position *j*.

In both *Oxytricha* and *Stylonychia*, the shortest introns (30–45 bp) tend to have strongest splice sites among other introns of these organisms. In *Tetrahymena*, the strongest 3' and 5' splice sites are found in introns with the length in the range 45–70 bp. These observations are in agreement with the intron-definition mechanism being predominantly used for splicing of short introns [25]. A reverse pattern has been observed for 24–45 bp long introns of *Tetrahymena*, that tend to have weaker splice sites. Similar to other introns of *Tetrahymena*, they possess the same conserved nucleotides at their 5' and 3' ends, (GT…AG), but are associated with gaps in protein alignments with orthologs from other studied species, indicating that these introns could be gene annotation artifacts (S2 Fig).

Unlike other species, *Ichthyophthirius* demonstrates no dependency between the strength of the splice sites and the intron length (Fig 4B).

### Frequencies of in-frame stop codons in ultra-short introns

We next analyzed frequencies of in-frame stop codons in 3n introns in comparison with 3n±1 introns across the studied species. The background frequencies of in-frame stop codons were generated by Monte-Carlo simulations as described in Methods (“Estimation of in-frame stop-codon frequencies in introns”).

**Fig 5:**
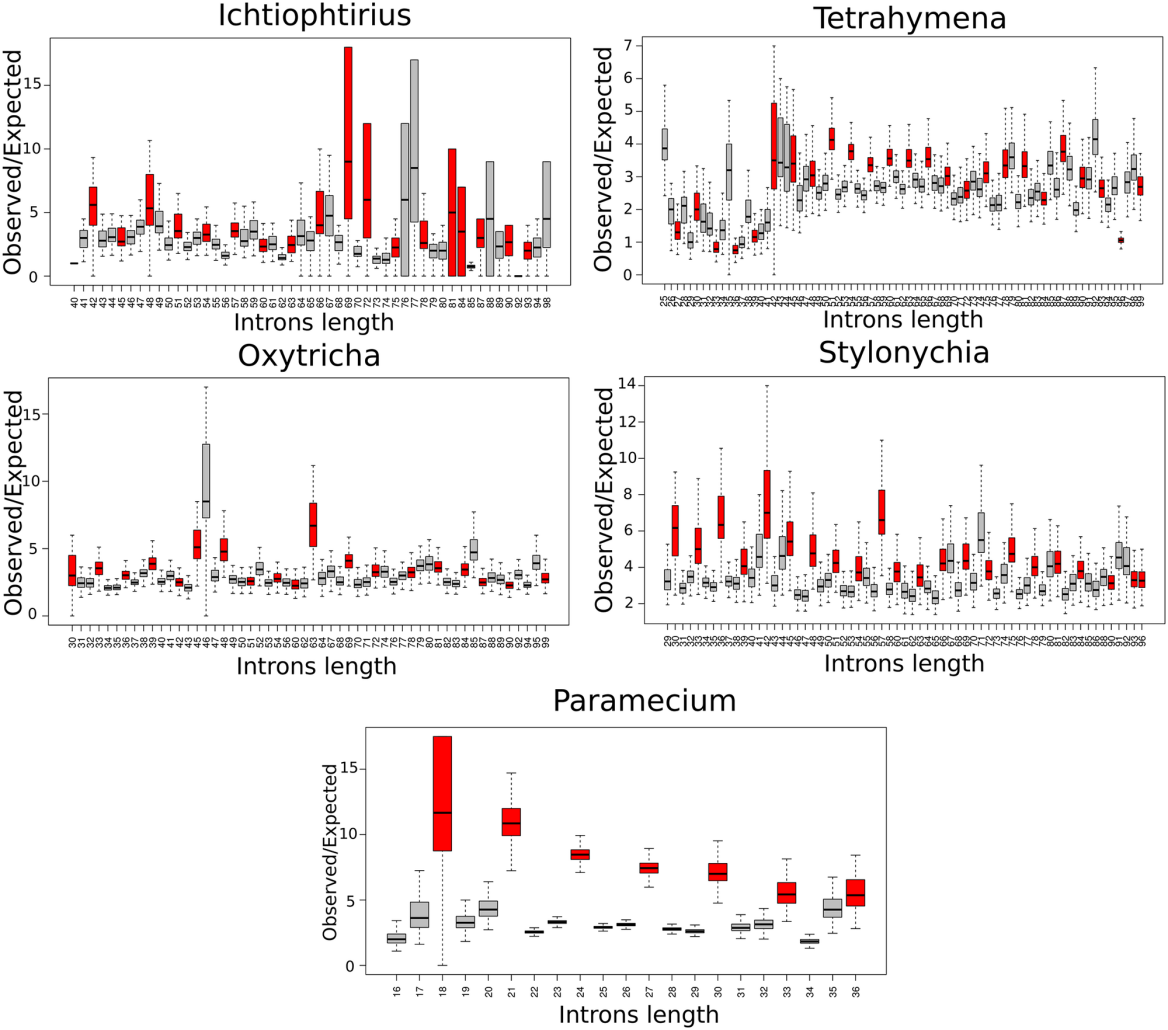
Enrichment of in-frame stop codons in introns of *Paramecium*, *Tetrahymena*, *Ichthyophthirius*, *Oxytricha*, and *Stylonychia*. Red and gray boxplots designate O/E values for 3n and 3n±1 introns, respectively. Boxplots were obtained by combining O/E values from 1000 replicates. P-value of stop-codons enrichment was less than 10^-4^ (by Monte-Carlo 1000-fold simulations) for all introns of *Paramecium*, *Oxytricha*, *Stylonychia*, 41–100 bp long introns of *Tetrahymena*, and almost all introns of *Ichthyophthirius*.

Our results indicate that in-frame stop-codons in short 3n introns are enriched in all studied ciliates. The ratio of the observed and expected numbers of 3n introns with in-frame stop codons was the highest in all introns of *Paramecium*, 50–70 bp introns of *Tetrahymena*, 30–40 bp introns of *Oxytricha*, and 30–70 bp introns of *Stylonychia* (Fig 5), overlapping with the regions of significant 3n introns underrepresentation in these species. Again, in contrast to other studied ciliates, in *Ichthyophthirius* 40–60 bp 3n introns were only slightly enriched in in-frame stop codons, compared to 3n±1 introns. The observed underrepresentation of 3n introns and their enrichment in in-frame stop-codons indicate that splicing of the short introns is an error-prone mechanism in Ciliates, leading to the inclusion of introns into mRNAs that should be degraded by the nonsense-mediated decay.

### Identification of conserved introns

**Fig 6:**
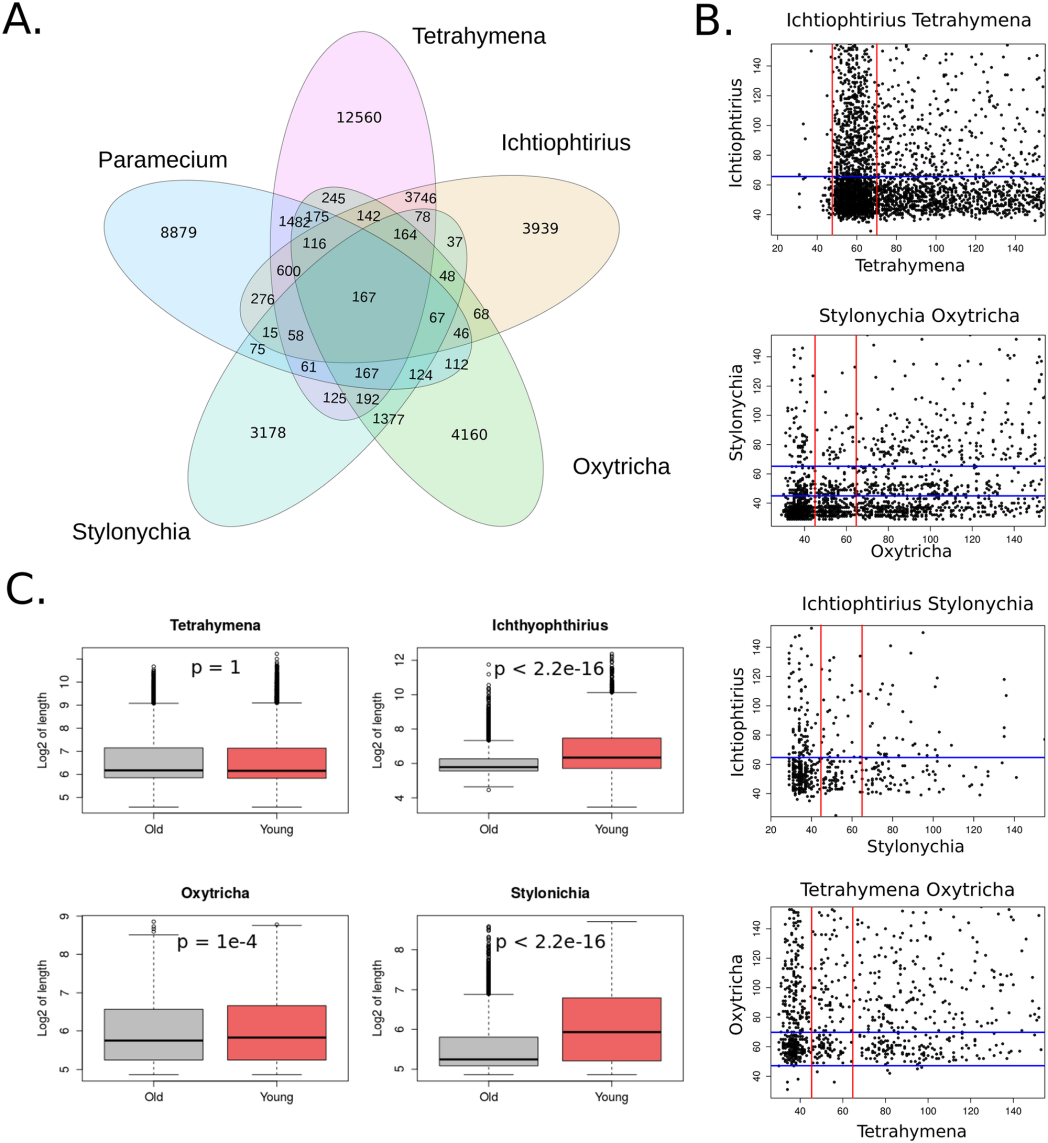
**A. The numbers of introns conserved between the orthologs of *Paramecium*, *Tetrahymena*, *Ichthyophthirius*, *Oxytricha*, and *Stylonychia*. B. Scatter-plots of orthologous introns length of *Tetrahymena*, *Ichthyophthirius*, *Oxytricha*, and *Stylonychia***. The intron length range up to 150 bp is shown. Red and blue lines separate classes of the intron lengths. **C. L distributions of the conserved and young introns in orthologous genes for *Tetrahymena*, *Ichthyophthirius*, *Oxytricha*, and *Stylonychia* (in the log2 scale)**. Conserved introns are significantly shorter than young introns in *Ichthyophthirius*, *Oxytricha*, and *Stylonychia* (by the Wilcoxon rank sum test, p-value < 2.2×10^-16^, < 10^-4^, and < 2.2×10^-16^, respectively).

Conserved introns were identified as described in Methods (“Identification of conserved introns”). As expected, given the branching order in the phylogenetic tree (Fig 1), the largest number of common introns was found in two pairs of the most closely related species, *Tetrahymena*/*Ichthyophthirius* (5022 introns), and *Oxytricha*/*Stylonychia* (2266 introns) (Fig 6). Of note, however, is that the *Stylonychia* genome annotation was made using the *Oxytricha* annotation as a model, and thus the conservation results might be slightly biased for these species. *Paramecium* had most of the common introns with *Tetrahymena* and almost two-fold fewer with *Ichthyophthirius* and *Oxytricha*. In total, 167 intron positions were conserved between all studied species. In general, 28%, 37%, 59%, 43%, and 46% of intron positions were conserved in at least one of the remaining species for *Paramecium*, *Tetrahymena*, *Ichthyophthirius*, *Oxytricha*, and *Stylonychia*, respectively.

The numbers of introns in orthologous genes were moderately correlated between *Oxytricha* and *Stylonychia* (0.52, Spearman's rank correlation coefficient, SCC), and between *Tetrahymena* and *Ichthyophthirius* (0.66, SCC); a weak correlation was observed between the numbers of introns in orthologs of *Paramecium* with either *Tetrahymena* or *Ichthyophthirius* (0.38 and 0.31 SCC, respectively).

Contrary to our expectations, we found that the length of conserved introns did not correlate between both closest and distant organisms (Fig 6). The intron length classes were also independent of each other between the species (S3 Fig). Comparing length distributions of the conserved (old) and not conserved (young) introns in orthologs of the studied species, we found that old introns tend to be significantly shorter than young introns in *Ichthyophthirius*, *Oxytricha*, and *Stylonychia*, but not in *Tetrahymena* (Fig 6).

## Discussion and Conclusions

The first distinctive feature of ciliate introns is the multimodality of their length distributions and the presence of intron length classes that are different between oligohymenophoreans and spirotricheans. These features of the ciliate introns were initially identified when annotating their genome sequences [5, 6, 8], but not yet explored in detail.

The bimodality of intron length distributions has been observed in many eukaryotic species. Two classes of introns, with a high peak of short (termed 'minimal length') introns and a much flatter peak of longer introns, ranging up to thousands of base pairs are present in humans [26, 27]. The functional link between intron length classes and gene expression suggests that introns tend to be smaller in highly expressed genes to provide for better transcriptional efficiency [27, 28]. The effect of intron length is even more important when only one copy of the gene is expressed, e.g. for imprinted genes [29] or genes expressed in a haploid pollen [30].

The bimodality of intron lengths is also associated with two major modes of recognition and pairing between adjacent splice sites, so-called “intron definition” and “exon definition” models [25, 31]. In vertebrate genomes, recognition of short introns primarily relies on intronic motifs such as splice sites, which are paired across the introns during excision, whereas the excision of longer introns is additionally supported by cis-acting elements yielding splice-site pairing across the exons [32]. In lower eukaryotes, where the genome architecture is characterized by small introns and long exons, the intron definition mechanism is probably predominant [32]. In addition to the splice motifs, intron length is critical for splicing efficiency in the organisms with the shortest introns, such as nucleomorphs. While the majority of introns of these species are 18–20 bp long and are efficiently removed, 21–27 bp introns show a substantial drop in their splicing efficiency [15]. It has been suggested that due to intron size and, consequently, information content reduction, intron size itself becomes a recognition signal, restricting the spliceosome ability to recognize introns over a certain size range [15].

Interestingly, in both *Tetrahymena* and *Ichthyophthirius*, bimodal intron length distributions are observed, suggesting that, similar to higher eukaryotes, they may possess two different mechanisms of the intron recognition and splicing, whereas, *Paramecium* has likely reduced or completely lost the exon definition mechanism. In general, the variability of the maxima of the intron length distributions and length classes across the studied oligohymenophoreans suggest that the evolutionary constrains imposed on intron length vary between the species possibly leading to the existence of several alternative mechanisms of introns recognition in these species. To our knowledge, spirotricheans are the only organisms with trimodal intron length distributions. Although the extent of this phenomenon in other species is not known, this could be evolutionarily associated with genome rearrangements and extensive IES removal during the MIC-to-MAC genome transition.

In most intron classes of all studied organisms, the intron length distributions had a deficit in introns whose length is divisible by three (3n introns). Here, we show that 3n introns are significantly underrepresented in short introns of *Tetrahymena* and *Ichthyophthirius*, ultra-short introns of *Oxytricha*, and both ultra-short and short introns of *Stylonychia*. The results indicate that 3n introns are under the strongest evolutionarily pressure in *Paramecium* [20], *Tetrahymena*, and *Stylonychia*, whereas in *Oxytricha* and *Ichthyophthirius* they are significantly underrepresented only among short introns. Then we analyzed frequencies of in-frame stop codons in introns of the studied species and found that 3n introns were enriched in stop codons in those intron length classes, where 3n introns were significantly underrepresented. Similar to the previous result, the pattern was most pronounced in three organisms, *Paramecium*, *Tetrahymena*, and *Stylonychia*. Further analysis of splice motifs at the introns termini revealed that short introns of *Tetrahymena* and ultra-short introns of *Stylonychia* and *Oxytricha* tended to have stronger splice sites than long introns of the respective species, suggesting a selection towards improved site-specific recognition and effective splicing of short introns.

Different properties have been observed for ultra-short introns of *Tetrahymena*. Unlike short and ultra-short introns of *Tetrahymena* and *Oxytricha*/*Stylonychia*, respectively, they are significantly overrepresented among 3n introns, and ultra-short 3n introns are depleted in in-frame stop-codons. These ultra-short introns also tend to have weaker splice sites. Hence, in the case of their retention, these introns would cause frequent read-through during the translation of a mis-spliced mRNA. However, we found that only a few ultra-short introns of *Tetrahymena* are conserved, and that ultra-short introns are associated with insertions in alignments with orthologous proteins of other studied species. The latter indicates that these introns are probably annotation artifacts, which would also explain their low conservation and weak splice sites.

There are several distinctive features between the studied oligohymenophoreans (*Paramecium*, *Tetrahymena*, *Ichthyophthirius*) and spirotricheans (*Oxytricha* and *Stylonychia*). They possess different numbers of intron classes (two and three, respectively). Oligohymenophoreans have two conserved nucleotides at their intron termini (5'–GT…AG–3'), whereas spirotricheans have three additional conserved nucleotides at the introns boundaries (5'–GTAAG…TATAG–3') and have stronger splice sites. Introns of oligohymenophoreans have two peaks of the introns densities, at 5' and 3' ends of genes, whereas introns of *Oxytricha* and *Stylonychia* tend to occur at the 5' end of a gene.

Introns of protein-coding genes are also unevenly distributed towards the 5′ ends of genes in *Saccharomyces cerevisiae* [33] and other intron-poor genomes, but are evenly distributed within coding sequences of genes in intron-rich genomes [34]. The explanation of this trend suggests the preferential loss of introns from the 3' regions of genes, possibly due to 5'-truncated transcripts subject to reverse transcription and causing intron loss via homologous recombination [33, 35, 36]. The model assumes formation of a stem-loop between DNA and cDNA, with intron loss dependent on the length of a 3' terminus folding back during homologous recombination and yielding introns losses from either the 3' end or the middle of a gene [37]. While spirotricheans demonstrate a strong 5' bias of the introns density, which is also observed in other intron-poor organisms, oligohymenophoreans show a different trend, suggesting an alternative or complementary mechanism of introns loss in oligohymenophoreans presumably from middle regions of the genes. The observed differences in the characteristics of short introns between oligohymenophoreans and spirotricheans may derive from peculiarities of their MAC genome structure and the mechanisms of its formation, which, in spirotricheans, involve extensive genome re-organization and scrambling, at some part mediated by RNA [13].

We found that a substantial fraction of introns is conserved in orthologs, ranging from 28% in *Paramecium* and 59% in *Ichthyophthirius*, reflecting the phylogenetic distance between the studied species. However, despite that, introns lengths and the introns length classes are not correlated between either distant or close species. The latter indicates that, unlike intron position in a gene, intron length is subject to fast changes in Ciliates.

In summary, our study provides a detailed characterization of the introns in two classes of Ciliates and highlights some of their distinctive properties, which, together, indicate that splicing spellchecking is a universal and highly evolutionary important process in the biogenesis of short introns in various representatives of Ciliates.

## Methods

### Data sources

Genome, proteome, and annotation data of *Paramecium tetraurelia* was obtained from [5, 38] (“Ptetraurelia_genes_v1.fasta”,“Ptetraurelia_peptides_v1.fasta”, and “parameciumDB.gff3” files); *Tetrahymena thermophila*, from [6, 39] (“T_thermophila_oct2008_gene.fasta”, “T_thermophila_oct2008_proteins.fasta”, and “T_thermophila_dec2011.gff3” files); *Oxytricha trifallax*, from [8, 40] (“Oxytricha_trifallax_022112_assembly.fasta”, “Oxytricha_trifallax_022112_aa.fasta”, and “Oxytricha_trifallax_022112.gff3” files); *Ichthyophthirius multifiliis*, from [7, 41] (“img1_0407.assembly.fsa”, “img1_0407.aa.fsa”, and “img1_0407.gff3” files); *Stylonychia lemnae*, from [9, 42] (“stylo_assembly.fa”,”stylo_protein.fa”, and”stylo.gff3” files).

### Calculation of 3n introns underrepresentation

For the intron length distributions of *Tetrahymena*, *Ichthyophthirius*, *Oxytricha*, and *Stylonychia*, we determined intervals of the intron length, where 3n introns are underrepresented, as follows: starting from the minimum intron length of the organism up to 100 bp, with a 3b-long sliding window, introns of the corresponding range of lengths were selected. Next, the proportion of 3n introns within each window was calculated. Underrepresentation of 3n introns in a window was calculated as the ratio between the observed proportion of 3n introns and the expected one, which is 0.33, calculated as described above. Next, we combined 3 bp long intervals, where 3n were underrepresented, into longer continuous intervals. Similarly, we calculated the proportion of 3n introns in these longer intervals and calculated significance with the two-sample proportion test between the observed proportion of 3n introns in the longer intervals, and the expected one, equal to 0.33.

### Estimation of in-frame stop-codon frequencies in introns

In order to estimate the enrichment, the background (expected) frequencies were generated as follows: for each length of introns, starting from the minimal intron length of the organism, through 100 bp, a random set of the same size, with the nucleotide frequencies as in the corresponding introns, of the length N–4 was generated 1000 times, where N is the intron length and 4 corresponds to two absolutely conserved dinucleotides at the intron termini. Next, the ratio between the numbers of the observed and randomly generated introns with in-frame stop-codons (TGA) was calculated. P-values were calculated as the number of times from 1000 replications when the expected ratio was larger or equal to the observed one.

### Construction of COGs (Clusters of Orthologous Groups)

First, two rounds of psi-BLAST protein search were performed, with and without low-complexity filtering and composition-based statistics (“psiblast -comp_based_stats F -seg no” and “psiblast -comp_based_stats T -seg yes”), between putative proteins of *Paramecium*, *Tetrahymena*, and *Ichthyophthirius*. Next, COGs (Clusters of Orthologous Groups) were constructed by the COGtriangles software (Update 20.04.12 [43]) and assignment of *Oxytricha* and *Stylonychia* putative proteins to these COGs was performed by COGnitor [44, 45] after running two passes of psi-BLAST with and without low-complexity filtering. Further, in-paralogs were filtered out, and one-to-one best hits between the organisms were selected using the best psi-blast (for *Paramecium*, *Tetrahymena*, and *Ichthyophthirius*) and Cognitor (for *Oxytricha* and *Stylonychia*) scores.

### Identification of conserved introns

Conserved introns were identified by the following algorithm: (1) protein sequences of orthologous genes in the same COG cluster were aligned with the “muscle” R package [46]; (2) for each intron position, 5 bp boundary regions at each side were selected; (3) for the alignment in the selected region, two parameters were estimated: *a*, the total number of gaps in the aligned proteins, and *b*, the distance between two introns positions in the alignment. Intron insertion was considered to be conserved if *a* ≤ 3 and *b* ≤ 2. The algorithm is schematically outlined in S1 Fig.

### Statistical analysis and software

Locations of introns and their sequences were obtained from the annotation and genome sequence data with custom scripts written in R. Sequence logos were constructed using the WebLogo on-line tool [47]. Decomposition of introns length distributions into a sum of normal distributions was performed with the standard Expectation Maximization algorithm for normal mixtures implemented in “mixtools” R package [48]. All statistical analyses were performed in the R programming language.

## Supporting Information

S1 Fig The pipeline for the identification of conserved introns. (1) Protein sequences of orthologous genes in the same COG cluster were aligned with “muscle” R package [40]; (2) for each intron position, 5 bp at each side were selected; (3) for alignment in a selected region, two parameters were estimated: number of gaps in both aligned proteins (a) and distance between two intron positions in an alignment (b). An intron position was considered to be conserved if a≤3 and b≤2.
(TIFF)

S2 Fig Frequencies of gaps around intron positions in protein alignments of *Tetrahymena* and *Ichthyophthirius* orthologs. For comparison, two intron groups are shown: ultra-short (20–45 bp) and short (45–70 bp). In total, 254 ultra-short and 5199 short introns were considered. Orthologs were taken from COGs, which were constructed as described in Methods (“Construction of COGs”).
(TIFF)

S3 Fig Concordancy between the classes of conserved introns in orthologs of *Tetrahymena*, *Ichthyophthirius*, *Oxytricha*, and *Stylonychia*. The percent of the total number of conserved introns in a pair of species is indicated in each cell.
(TIFF)

S4 Fig Densities of introns along the genes. Genes containing at least three introns were selected and divided into three equal intervals; then numbers of introns in each interval were calculated.
(TIFF)

S1 File Coordinates of introns in genes (or contigs) and proteins of *Paramecium*, *Tetrahymena*, *Ichthyophthirius*, *Oxytricha*, and *Stylonychia*. The introns coordinates are given in numbers of base pairs and numbers of aminoacids for genes and proteins, respectively.
(ZIP)

S2 File Conserved introns. Tables include COG IDs, protein IDs, and corresponding intron IDs. Conserved introns were identified as described in Methods (“Identification of conserved introns”).
(ZIP)

S3 File Clusters of orthologous groups. (TXT)
