# Supplementary figures and images for: Evolution of the Exon-Intron Structure in Ciliate Genomes
Source: PLoS One. 2016 Sep 7;11(9):e0161476. doi: 10.1371/journal.pone.0161476 (PMC5014332; doi:10.1371/journal.pone.0161476)

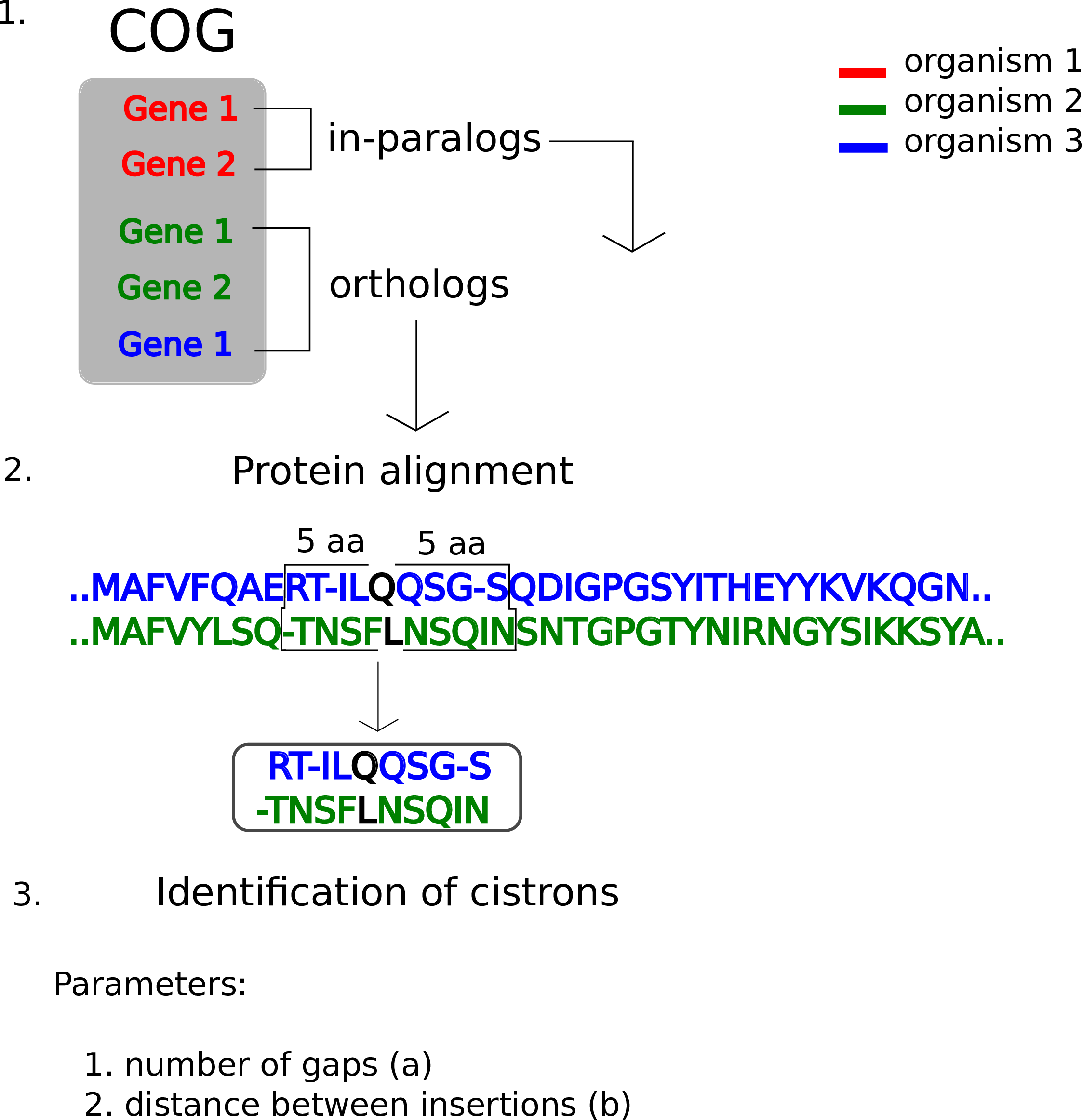

Supplement: S1 Fig — (1) Protein sequences of orthologous genes in the same COG cluster were aligned with “muscle” R package [40]; (2) for each intron position, 5 bp at each side were selected; (3) for alignment in a selected region, two parameters were estimated: number of gaps in both aligned proteins (a) and distance between two intron positions in an alignment (b). An intron position was considered to be conserved if a≤3 and b≤2. (TIFF) [file pone.0161476.s001.tiff]

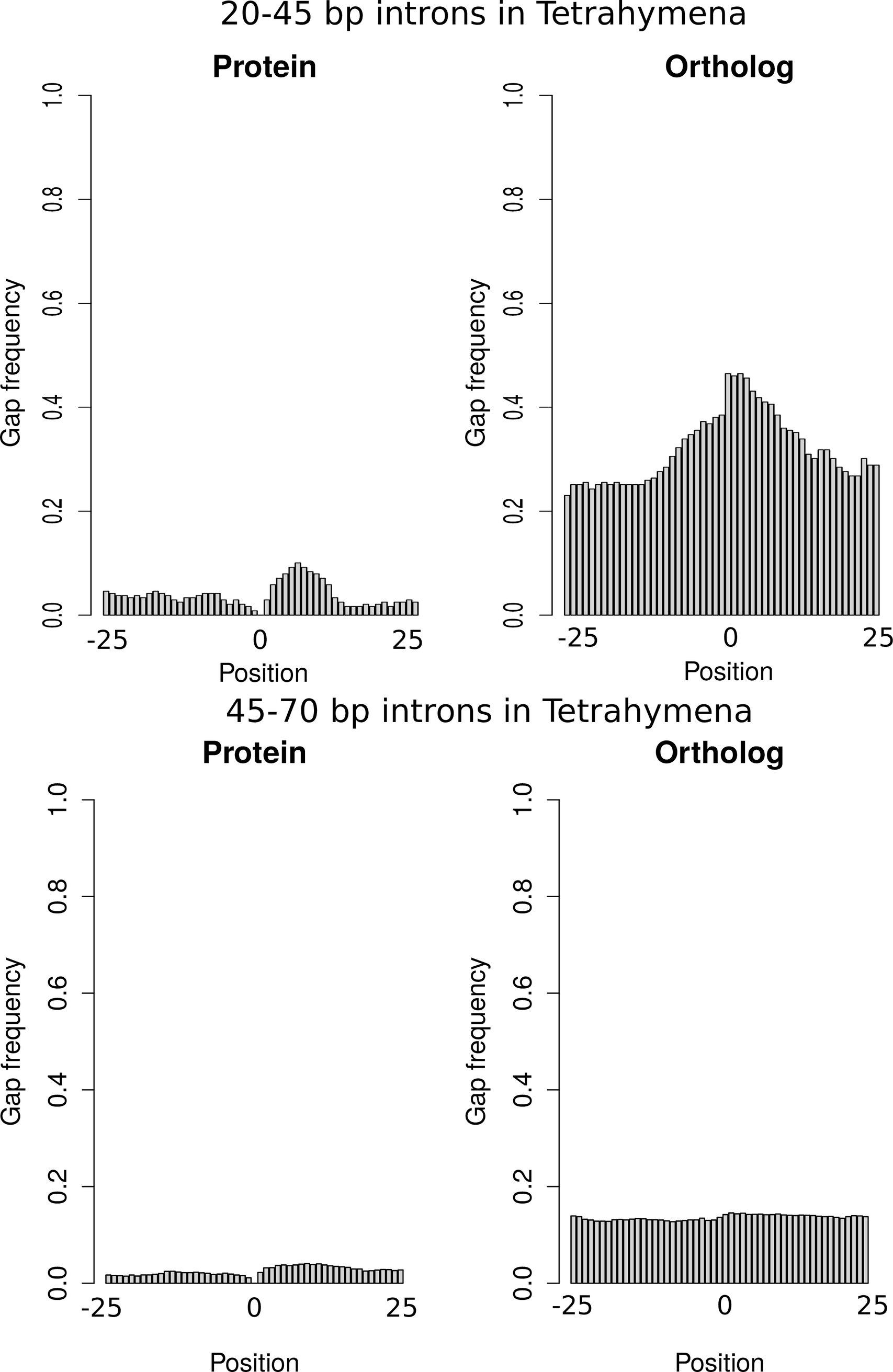

Supplement: S2 Fig — For comparison, two intron groups are shown: ultra-short (20–45 bp) and short (45–70 bp). In total, 254 ultra-short and 5199 short introns were considered. Orthologs were taken from COGs, which were constructed as described in Methods (“Construction of COGs”). (TIFF) [file pone.0161476.s002.tiff]

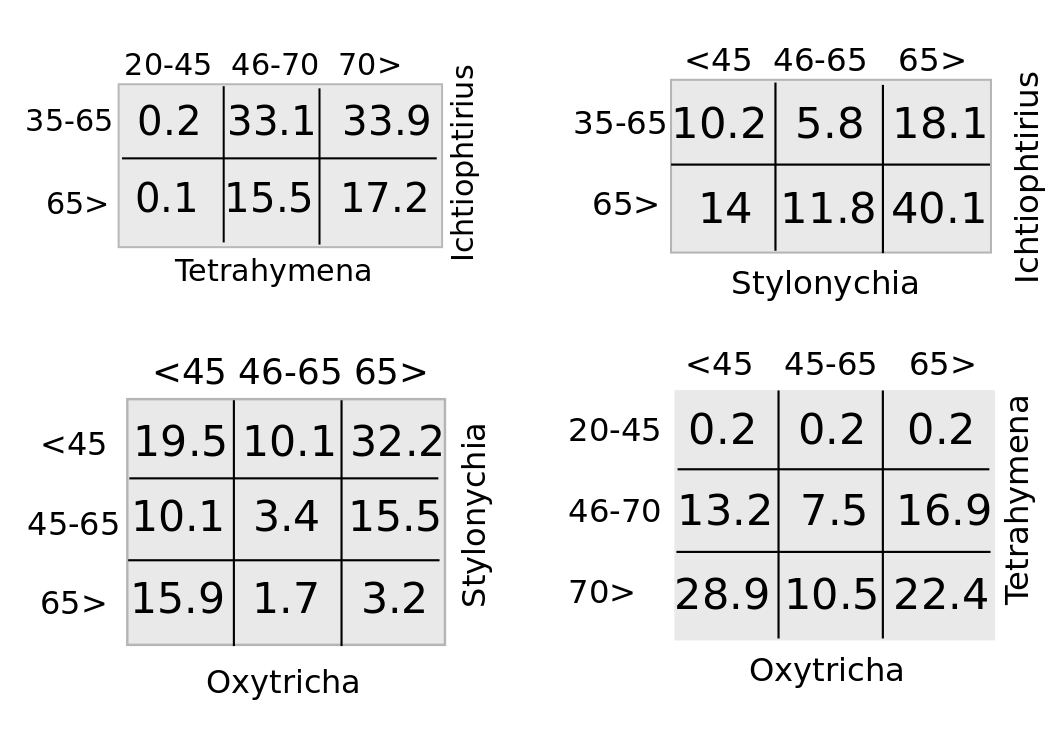

Supplement: S3 Fig — The percent of the total number of conserved introns in a pair of species is indicated in each cell. (TIFF) [file pone.0161476.s003.tiff]

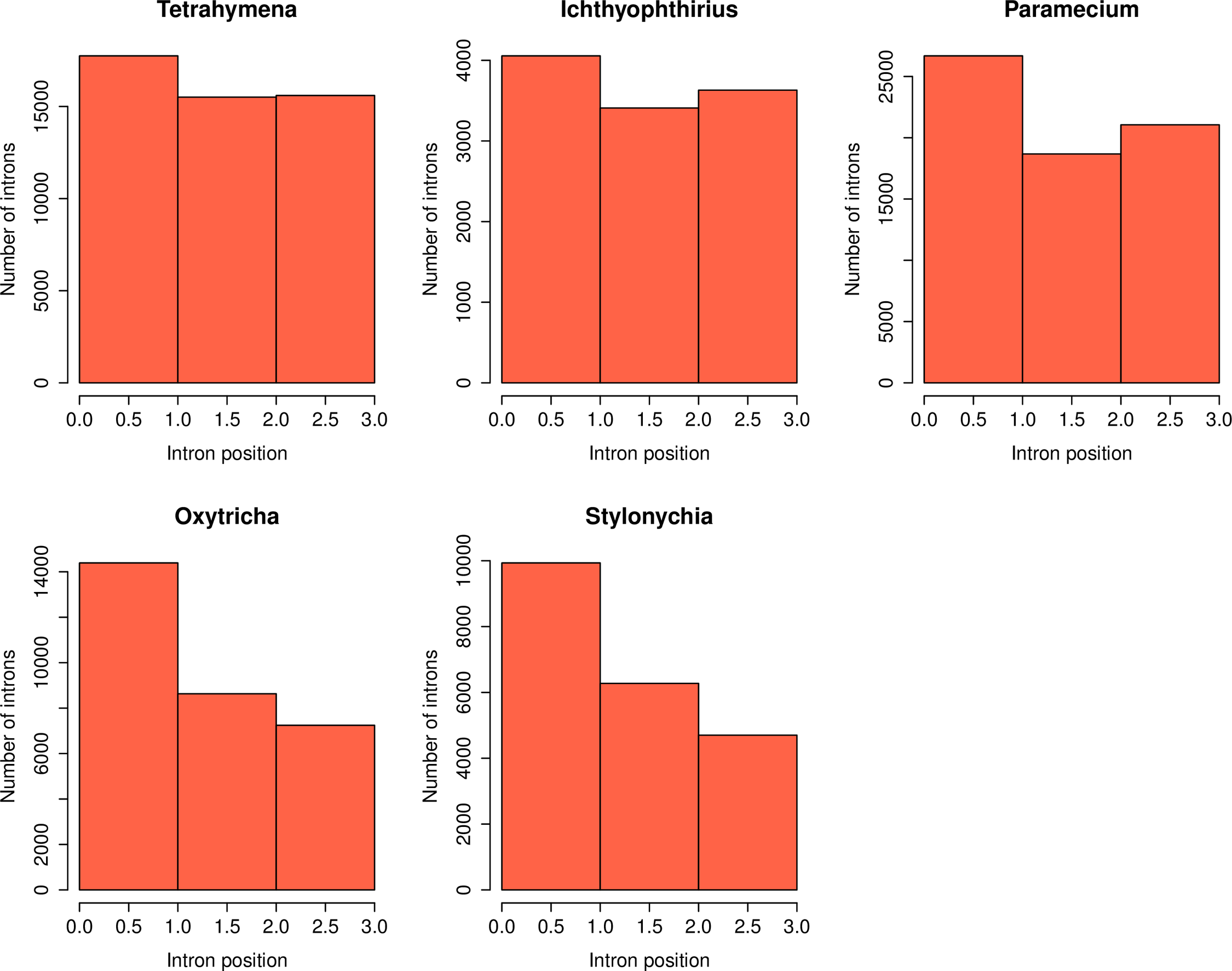

Supplement: S4 Fig — Genes containing at least three introns were selected and divided into three equal intervals; then numbers of introns in each interval were calculated. (TIFF) [file pone.0161476.s004.tiff]
